# AN EMOTION REGULATION THERAPY FOR LATER-LIFE PAIN: EVIDENCE OF EARLY TREATMENT EFFECTS

**DOI:** 10.1093/geroni/igz038.2317

**Published:** 2019-11-08

**Authors:** Emily Petti, Dimitris Kiosses, Lisa Ravdin, Charles Henderson, Lauren Meador, Julianna Maisano, Cary Reid

**Affiliations:** 1 Weill Cornell Medicine, New York, New York, United States; 2 Cornell University, Ithaca, New York, United States

## Abstract

Chronic pain (CP) is a common, morbid, and costly disorder in older adults. Guidelines encourage clinicians to employ non-pharmacologic therapies for its management, but current psychological interventions (e.g., CBT for pain) have modest treatment benefits and their effects are largely unknown in older cognitively impaired adults. We developed PATH-Pain, an emotion regulation therapy focused on reducing negative emotions and augmenting positive emotions. PATH-Pain is appropriate for use by older adults with CP, negative emotions, and a wide range of cognitive functioning. Treatment consists of 8 weekly individual sessions followed by 4 monthly booster sessions. One hundred older adults (ages 60+) with CP (≥ 3 months) and at least mild-to-moderate levels of negative emotions (per the Positive and Negative Affect Schedule) were randomized to receive PATH-Pain versus Usual Care (UC). Cognitive screening revealed that 44 participants were cognitively intact (Montreal Cognitive Assessment (MoCA) score ≥26), while 56 evidenced mild-to-moderate cognitive impairment (MoCA=16-25). Participants completed follow-ups at 5 (n=89) and 10 weeks (n=84), while 24-week assessments are ongoing. Examination of the treatment × time interaction in a repeated-measures mixed model indicate the presence of treatment effects. PATH-Pain (vs. UC) participants experienced significant reductions in pain intensity (p<0.044) and pain-related disability (p<0.003). Reductions in pain-related disability score were more pronounced among cognitively impaired individuals. The PATH-Pain group also demonstrated significant reductions in emotional suppression (p<0.019) and depression (p<0.009) scores. These results suggest that PATH-Pain is an effective treatment for the management of pain in cognitively intact and cognitively impaired older adults.

